# Meeting of Ex-Confederate Army and Navy Officers

**Published:** 1875-10

**Authors:** 


Meeting of Ex-Confederate Army and Navy Medical Officers.—We find the following notice in the Virginia Medical Monthly for September:

The Association of Medical Officers of the Confederate Army and Navy will convene at Richmond at 11 A. M., Tuesday, October 10th, 1875. All communications for this Association should be addressed to the President, Dr. S. P. Moore, Richmond, Va., or to the Secretary, Dr. S. H. Stout, Atlanta, Ga.

Every effort will be made to secure the usual reduced fare over railroads, boat lines, etc., which, as far as practicable, will be announced in the forthcoming circular. It is a pleasure, however, to announce at this early day that the proprietor of St. James Hotel—*one of the very best in the State in every respect*—has agreed to charge the members with their families, who may attend, only *two dollars* per diem during the period in which either or both of these Societies may be in session.

